# Multifunctional transition and temperature-responsive contact lenses

**DOI:** 10.1038/s41377-023-01304-1

**Published:** 2023-11-13

**Authors:** Ahmed E. Salih, Haider Butt

**Affiliations:** Department of Mechanical Engineering, Khalifa University, Abu Dhabi, UAE

**Keywords:** Optical materials and structures, Optical sensors

## Abstract

Smart contact lenses have recently gained traction due to their functionalization as noninvasive diagnostic and therapeutic wearables that can address several ocular diseases. Herein, multifunctional contact lenses exhibiting UV-transition and temperature-responsive capabilities were developed utilizing chromogenic materials that were integrated simultaneously into poly(2-hydroxyethyl methacrylate) (pHEMA) contact lenses. The functionalities of the contact lenses were optically evaluated in both their activated and non-activated states. Transition contact lenses offered excellent UV and blue light blocking capabilities (~45%) at their inactive states. When activated via UV exposure, the transparent lenses darkened instantaneously and absorbed portions of the visible light spectrum. The absorption intensity and transient discoloration of the transition lenses relied primarily on the utilized photochromic material. Likewise, the temperature-responsive contact lenses exhibited distinct colorimetric variations in response to temperature changes within the physiological range (33–38 °C). The maximum sensitivity of the thermochromic lens was 8% transmitted light per Celsius degree shift. Physiochemical and morphological analysis indicated the adequacy of the contact lenses. Hence, the multifunctional contact lenses can be deployed as smart wearables to manage ophthalmic deficiencies that are deterred by UV radiations and variations in ocular surface temperature.

## Abstract (web summary)

We report fabrication of multifunctional transition and temperature-responsive contact lenses that undergo colorimetric switch in response to UV light and physiological temperature changes.

## Graphical abstract

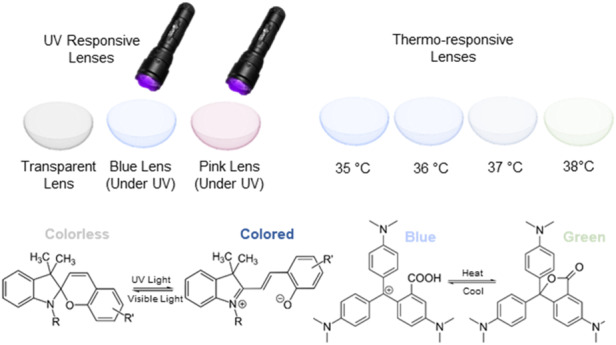

## Introduction

Ocular deficiencies are becoming more prevalent than ever due to lack of necessary ophthalmic diagnostic and monitoring methods. Nonetheless, recently developed therapeutic and diagnostic contact lens devices have had huge success in treatment and management of ocular diseases^1–9^.

For instance, cataract is one the most prominent vision impairment deficiencies, reportedly affecting 94 million people and causing more than 10 million annual corrective surgeries^10^. Excessive exposure to ultraviolet (UV) light has been reported as a leading cause of cataract and other ocular deficiencies like age-related macular degeneration (AMD), photokeratitis, and eyelid malignancies^11^. As such, UV protective contact lenses developed by companies and researchers utilizing dyes, coatings, nanoparticles, and quasi-periodic structures have emerged as therapeutic wearables aimed to manage and, potentially, diminish development of cataract’s symptoms^12–15^. As per the UV filtered wavelengths by the contact lenses, they are classified into two different classes: (1) Class I block 99% of UVB (280–315 nm) and 90% UVA (316–380) and (2) Class II block 95% UVB and 70% UVA. Most commercial contact lenses are classified as Class II UV blockers^13^. Meanwhile, more recently, transition lenses have gained popularity as effective wearables to manage certain ocular conditions. Transition lenses darken when exposed to sunlight or UV light and turn clear indoors or in absence of UV. Most of these lenses are utilize photochromic molecules which are activated by exposure to UV. Although research on the perks of these lenses is still underdeveloped, few studies have shown their efficacy in managing AMD, dry eye syndrome, and photophobia^16–18^. However, the only commercial contact lens with transition properties currently available is the ACUVUE® OASYS with Transitions. The aforementioned contact lens has reportedly improved visual acuity in both bright and normal/low-light settings compared to a commercial UV blocking contact lens^19–21^. As predicted under bright light, the transition contact lenses significantly enhanced photostress recovery and glare discomfort^21^, yet unexpectedly, they were also better than UV blocking lenses under normal lightning. In fact, when tested in nighttime and with usage of digital devices, the transition contact lenses were superior as they improved clarity of vision and reduced squinting, emphasizing the technology’s ocular advantages over traditional UV blocking techniques.

Along with being deterred by UV radiations, ocular deficiencies like dry eye syndrome are significantly impacted by another vital ocular parameter, surface temperature^22,23^. Ocular surface temperature of healthy individuals ranges from 31 to 37 °C^22,24^. Variations in ocular temperature were linked to eye inflammations that aggravate dry eye, glaucoma, and diabetic retinopathy syndromes^22–28^. Hence, interest in real-time monitoring of ocular surface temperature using biosensing contact lenses has grown significantly over the past few years. For instance, Moreddu et al. developed scleral contact lens based on cholesteric liquid crystals embedded into etched micropatterns within the lens. The liquid crystals showed a reversible thermochromic behavior along with exhibiting a wavelength shift from 738 to 474 nm for the temperature range of 29–40 °C^23^. Likewise, Guo et al. introduced a facile approach to fabricate multifunctional smart contact lenses with an ultrathin MoS2 transistors-based serpentine mesh sensor system, whereby optical and continuous glucose and temperature monitoring data could be measured from the sensing platform. For temperature sensing, serpentine gold wires were incorporated into the outer surface of the corneal section of the contact lens, and the sensor showed adequate sensitivity of 0.94 Ω/°C^29^. Fabrication complexity of the aforementioned thermo-sensitive contact lenses diminishes their viability and makes them highly undesirable.

**Fig. 1:**
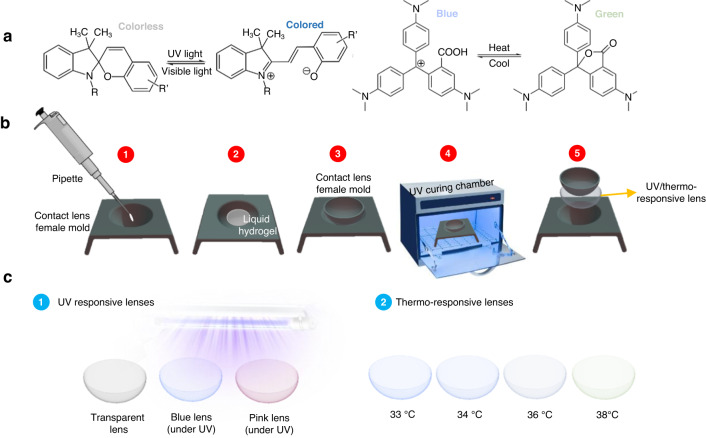
Fabrication of multifunctional transition and temperature sensitive contact lenses. **a** Chemical structure of photochromic and thermochromic powders (LEFT) spiropyran structured material (colorless) switching to merocyanine (colored) and (RIGHT) Fluoran leuco dyes at protonated and deprotonated states. **b** Schematic of the lenses’ fabrication process. **c** Multifunctional contact lenses under different stimuli

Herein, we fabricated simple low-cost multifunctional transition and temperature-sensitive contact lenses based on photochromic and thermochromic powders. Although these dyes were discovered decades ago, their utilization in applications like textiles, optics, smart wearables, and sustainable buildings is very recent^30–33^. Photochromic micro-powders are generally made of a core photochromic material like spirooxazine or spiropyran, which upon exposure to UV radiation isomerizes reversibly and forms a merocyanine structure (Fig. 1a)^34^. Spiropyrans and their derivatives are considered some of the most prominent types of photochromic materials owing to their isomerization versatility under different stimuli like light, temperature, and pH^35^. Under the functional stimulus, the C-O spiro bond undergoes cleavage, resulting in the formation of the merocyanine form. The reversible conversion involves a charge separation yielding an increase in the dipole moment from 4–6 D for spiropyrans to 14–18 D for merocyanine^35^. Furthermore, the core photochromic material is generally encapsulated within a melamine formaldehyde resin that provides prolonged stability for the powder. Likewise, thermochromic powders are made of temperature-responsive dyes or pigments like liquid crystals, leuco dyes, or inorganic pigments (e.g. titanium dioxide and iron oxide)^36^. As for fluoran leuco dyes, their protonated/deprotonated states in response to temperature changes dictate the arrangement of the molecule, hence, the powder’s resulting color^37^. Prior to incorporating the powders into contact lenses, their optical, material, and morphological properties were obtained. Similarly, the optical and physiochemical properties of the contact lenses, including transmission under visible light and UV light were recorded. Simple yet effective incorporation of these stimuli-responsive powders can pave the way for further fabrication of smart therapeutic contact lenses for management and prevention of various ocular diseases.

## Results

**Fig. 2:**
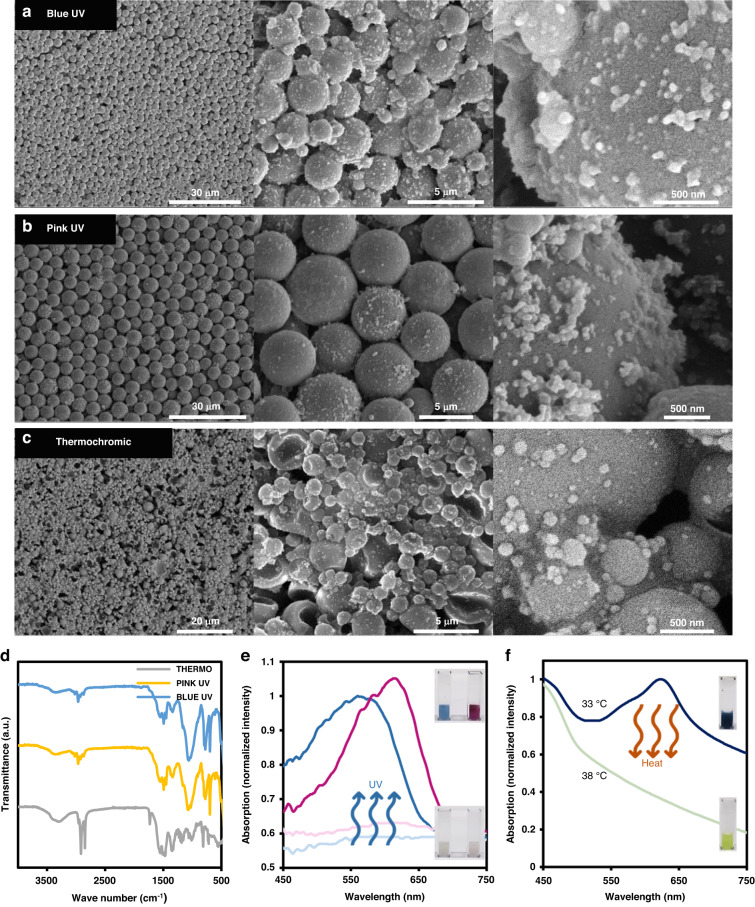
Powder characterization. SEM images at different magnifications of (**a**) Blue UV, (**b**) Pink UV, and (**c**) Thermochromic microcapsule powders. **d** FTIR spectra of the three powders. Optical absorption spectra of (**e**) UV and (**f**) Thermochromic powders, showing both inactive and active state responses based on triggering stimulus

The morphology, chemical composition, and optical properties of the thermochromic and photochromic microcapsule powders were initially characterized and are shown in Fig. 2. SEM images of the photochromic powders demonstrate the microcapsules’ spherical morphology. Few photochromic micro-powders exhibited slight distortions and disintegrated, from which their core-shell structures became apparent. Diameters of the blue and pink photochromic pigments were in the range of 1–3 µm and 3–8 µm, respectively. Likewise, thermochromic powders were also circular in morphology but with more frequent particle distortions, which results in slightly less stable structure. Thermochromic micro-powders’ diameters were also around 1–5 µm. FTIR spectra of the three powders are shown in Fig. 2d. Both photochromic powders had similar characteristic peaks. The peak at 3355 cm^−1^ is the stretching vibration peak of N-H and O-H bonds while peak values while the peak value at 2956 cm^−1^ indicates the asymmetric –CH stretching vibration of –CH_2_OH. The latter are characteristic peaks of urea formaldehyde resin^38^. Values at 2930 and 2872 cm^−1^ refer to the –CH_2_ asymmetric and symmetric stretching vibrations, which are characteristic peaks of alkyd resin^39^. The peak at 697 cm^−1^ refers to flexural vibration absorption in the C–H plane of the benzene ring while the peak at 3025 cm^−1^ is due to C-H stretching on the benzene ring. These peaks are characteristic of styrene maleic anhydride copolymer^39,40^. The thermochromic powder shares few similar characteristic peaks with the photochromic powders (2956, 2920, 2852, 1550, 1462, and 700 cm^−1^) indicating that the coating in both powders is quite similar. The presence of the sharp peak at 1731 cm^−1^ can be attributed to C = O stretching vibration of a lactone ring in the leuco dye^41^. Furthermore, optical absorption spectra of the powders during inactive and active states were recorded (Fig. 2e, f). The UV powders are slightly opaque. As such, when dissolved in DMSO, their solutions have high absorption (low transmission). Upon being exposed to UV, the solution containing the powders instantly turned colorful. Intensity increase was almost two-fold, indicating a strong vibrant coloration due to UV stimulus. Moreover, the thermochromic powders dissolved in DMSO were blue in nature at 33 °C; upon heating to 38 °C, their color turned light green, and their absorption spectra decreased, with the blue indicative dip at 625 nm completely disappearing. Intensity variations were also almost similar to the photochromic powders. It is worth mentioning that the temperatures at which the thermochromic powder starts and stops coloration were obtained experimentally by varying the solution’s temperature from 25 to 45 °C.

**Fig. 3:**
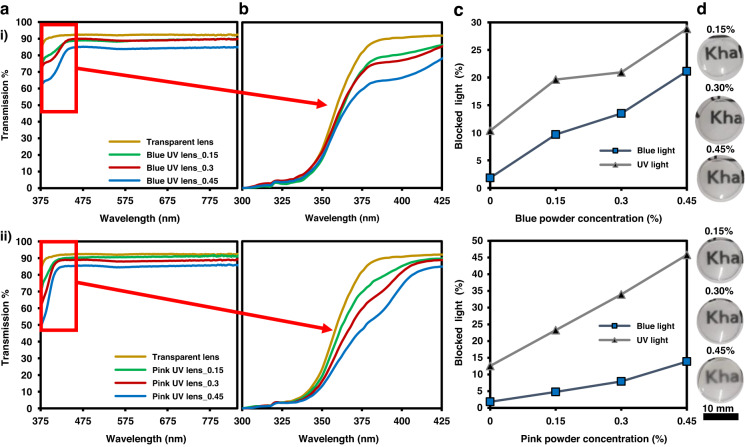
Optical properties of the transition contact lenses at their inactivated states. **a** Visible light and **b** UV light transmission spectra. **c** Percentage of blue light and UV light blocked as a function of powder concentration (Recorded at 400 and 375 nm for blue light and UV light, respectively). **d** Images of the transition contact lenses. i) Blue and ii) pink UV powder-containing contact lenses

Transmission spectra of the transition contact lenses at their inactivated states are shown in Fig. 3. Powder concentration was optimized to achieve adequate UV intensity responsiveness without compromising the transparency of the lenses. We found that the maximum concentration that can be added without significantly altering transmission is 0.5 wt%. As such, three different concentrations from each UV powder (blue and pink) were added to the contact lens’ solution, prior to polymerization. Overall transmission measurements of both sets of lenses were more than 85%, and the addition of the highest photochromic powder concentration reduced overall visible light transmission by 4%. However, addition of the photochromic powders improved the contact lens’ blue light and UV light filtering capabilities. Evidently, with the increase in powder concentration, the percentage of blocked UV and blue light also increased, yet, minimal presence of the powder nonetheless offered adequate filtering properties compared to the transparent untreated lens. Although both sets of lenses showed good UV and blue light absorption properties, the blue photochromic powder offered better blue light filtering capabilities than the pink powder, as it filtered out around a maximum of 20% of blue light (400 nm). Pink photochromic powders had significantly better UV protective capabilities as they exhibited a maximum absorption percentage of 45% at 375 nm. Also, transparency of the contact lenses with different concentrations is apparent from the digital images (Fig. 3d). Thus, even in their inactive states (under no UV illumination), these transition contact lenses can be used indoors as protective contact lenses against blue and minimal UV light.

**Fig. 4:**
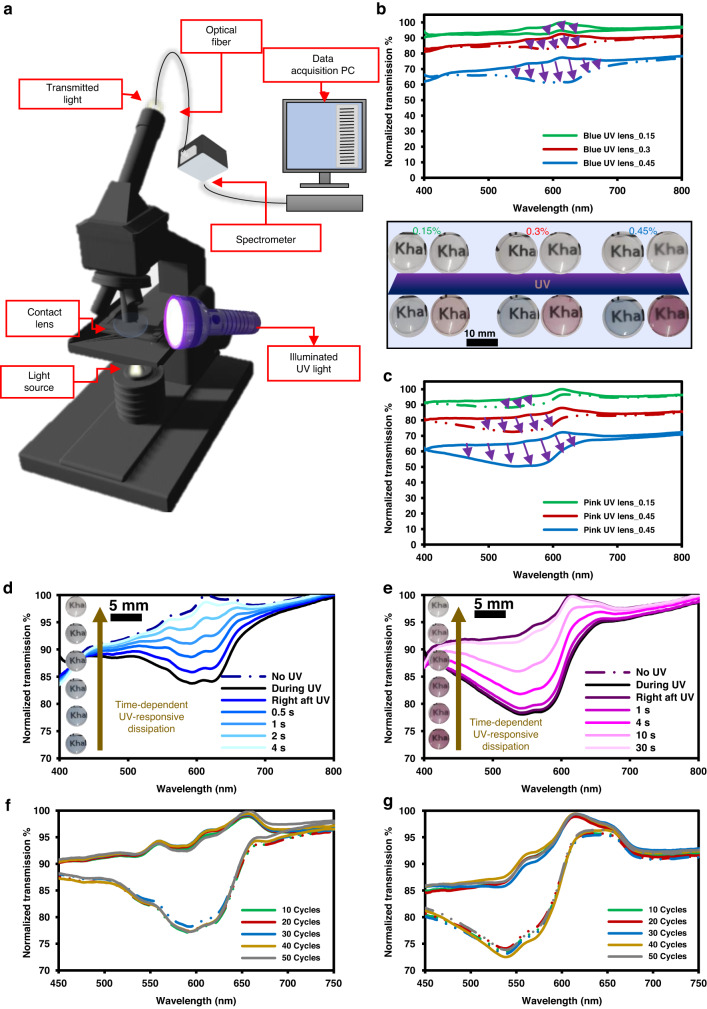
Optical properties of the transition contact lenses at their activated states (exposed to UV). Wavelength of illuminated UV light: 375 nm. **a** Schematic of the experimental setup for measuring the UV responsiveness of the contact lenses. Transmission spectra of (**b**) blue and (**c**) pink photochromic contact lenses with (solid line) and without (dashed line) UV light exposure. Inset shows digital images of the contact lenses before and after UV light exposure. Time-dependent transmission spectra and irradiation durability over 50 cycles of the highest concentrated (**d**, **f**) blue and (**e**, **g**) pink photochromic contact lenses

As a result of UV light exposure, transition lenses darken, allowing less light to pass through. The degree to which these contact lenses can darken (or respond to UV) can be measured by exposing them to UV light and recording the corresponding transmission spectra. Figure 4 shows the optical response of the transition contact lenses at their activated states, upon UV exposure. The experimental setup for measuring the lenses’ UV responsiveness consisted of a white light source integrated with an optical microscope that was connected to a UV–Vis spectrometer, and contact lenses were placed as shown in Fig. 4a. A UV torch (wavelength: 375 nm) was used to illuminate and activate the transition contact lenses. To achieve saturation of the photochromic powders, contact lenses were illuminated with UV light for 30 s. The UV responsiveness of both sets of lenses depended primarily on the concentration of the incorporated powder. Upon exposure to UV, the lenses darkened, consequently decreasing the transmission. The degree to which both sets of lenses exhibited maximum light absorption in response to UV varied between around 5–20%, for the utilized concentrations of 0.15–0.45%, respectively. Evidently, since the emitted colors were different, the visible light filtered wavelengths for each set of contact lenses were also distinct. As for the blue photochromic contact lenses, the most prominent filtered wavelengths were in the range of 550–650 nm, yet pink photochromic contact lenses absorbed more light between 500–600 nm. Transparency of the contact lenses was not significantly affected as a result of the photochromic activation. The digital images in Fig. 4 show that the transition contact lenses although darkened and became colored, they were still quite see-through. The transitional response of the contact lenses to the colored/darkened state upon UV exposure was instantaneous; however, the discoloration of both sets was variant. The blue transition contact lenses exhibited a fast response as 95% of discoloration was achieved in 4 s. The pink transitions lenses showed a much slower response as it retained its transparent state in more than 30 s.

Differences in switching dynamics between both types of lenses are primarily due to the spiropyran dye’s chemical structure and/or environment (solvent). More generally, photoactive molecules like spiropyrans have unique absorption wavelengths^35^; hence, the dyes within the powders could be slightly different. More specifically, the spiropyran’s solvent or chemical structure is the primary reason for changes in discoloration times along with intensity.

As for the spiropyran’s solvent, reducing its polarity diminishes the stability of merocyanine forms (ring-open forms), which shifts the equilibrium more towards colorless spiro isomer (ring-closed form)^42^. The latter results in a faster back-isomerization (reverse photochromism) to reach the more stable spiro form. Hence, the solvent used to dissolve the spiropyran’s dye might have been different for both pink and blue powders which could have been the reason for the difference in response time and intensity.

Similarly, the spiropyran used for both powders might also have different chemical structure. The two most common spiropyran dyes in literature are 5^SP^, indolinospironaphthopyran, and 6^SP^, indolinospirobenzopyran dye 6 possessing 6-nitro substitution^43^. The main difference in terms of chemical structure between both dyes is the replacement of the fused benzo ring in 5^SP^ with the 6-nitro group to yield 6^SP^^43^. Based on the literature, 5^SP^ dyes exhibit much faster reverse photochromism rates than 6^SP^ dyes, yet this results in more significant photochromic intensities for 6^SP^ dyes. The nitro substitution leads to a much more prominent photochromism compared to that of other indolinobenzopyrans that do not comprise nitro groups, subsequently yielding photochromic intensities that are atleast an order of magnitude more than that of other derivatives^43^. Another difference between both dyes relates to the their solvatochromism, color change due to solvent (polarity) variation. In fact, the introduction of the nitro group enhances the zwitterionic form of the merocyanine species, which shifts the dyes’ solvatochromism from positive to negative, thus causing changes in polarity and maximal absorbance trends^43^. For highly polar solvents, the 5^SP^ dyes have a maximum absorption at about 560–610 nm while the 6^SP^ dyes’ maximum absorption occurs at 530–560 nm^43^. As such and based on the photochromic optical properties of our lenses, we could distinctly categorize the blue and pink powders used, herein, as 5^SP^ and 6^SP^ dyes, respectively. The variation in the response time and absorption intensity is apparent between both transition contact lenses, allowing for user-specific customization. Moreover, to test the durability of the transition lenses, they were irradiated with UV light for 50 cycles, and their corresponding transmission spectra at transparent (spiro) and colored (merocyanine) states were recorded (Fig. 4). Throughout the 50 irradiation cycles, both sets of contact lenses exhibited analogous transmission spectra in their transparent and colored states. As such, they retained their intrinsic transparency and demonstrated consistent coloration intensity, which indicates their durability as transition contact lenses.

**Fig. 5:**
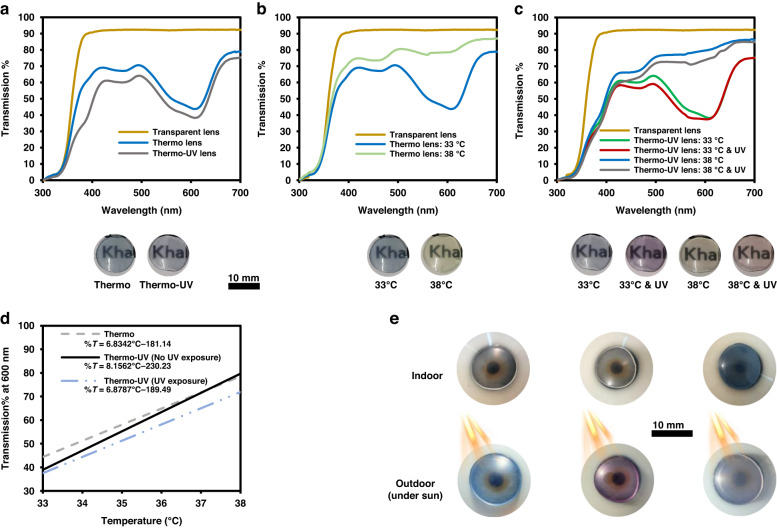
Optical properties of temperature sensitive and multifunctional contact lenses. Transmission spectra of (**a**) temperature sensitive and multifunctional lenses at room temperature, (**b**) temperature sensitive lens at 33 and 38 °C, and (**c**) multifunctional lenses at four different states: (1,2) At 33 °C with and without UV exposure. (3,4) At 38 °C with and without UV exposure. **d** Calibration curve (sensitivity of thermochromic response) of the temperature sensitive and multifunctional contact lenses (with and without UV exposure). **e** Digital images of the contact lenses indoor and outdoor showing their activated response at daylight

Temperature-responsiveness of thermochromic and multifunctional thermochromic and photochromic contact lenses was assessed (Fig. 5). To fabricate the multifunctional lenses, equal concentrations from both thermochromic and pink photochromic powders were added to the hydrogel solution prior to polymerization. Average transmission values of the thermochromic and multifunctional contact lenses were around 60–70%; the transmission dip at 500–650 nm is due to the apparent blue color of both lenses. The contact lenses were temperature-responsive between 33 and 38 °C, indicating their sensitivity at physiological conditions. Thermochromic contact lenses were blue at 33 °C, and upon heating to 38 °C, they turned light green, similar to their solutions (Fig. 2f). Changes in transmission intensity were also quite evident. Transmission at the dip exhibited a two-fold increase from about 40% to 80%. Similarly, the temperature-responsiveness of the multifunctional contact lenses was analyzed in two distinct illumination conditions: with and without UV exposure (Fig. 5c). Trends were analogous to those of the thermochromic contact lenses. Average transmission of the multifunctional lenses was ~60%; transmission at the dip increased by ~40% due to the increase in temperature. Under normal lightening, color alterations due to temperature were similar to that of the thermochromic contact lens (blue to light green), yet under UV exposure, the contact lens became pink at 33 °C and turned orange upon heating to 38 °C, at which both powders were activated. Hence, the UV-responsiveness and temperature-sensing capabilities were both incorporated and demonstrated.

The temperature responsiveness of both lenses was assessed by deducing the sensitivity around their functional temperatures (33–38 °C). The variations in transmission at 600 nm were used to assess the sensitivities as they were most pronounced in that wavelength. The thermochromic contact lens exhibited a change of around 6.8% in transmission as a result of one Celsius degree shift. The multifunctional contact lens, under no UV exposure, demonstrated a better temperature sensitivity than the thermochromic lens (8.1%T per °C), which is unexpected as the incorporation of UV-responsive materials was predicted to slightly impede the temperature responsiveness of the lens. Nonetheless, the temperature responsiveness of the multifunctional lens was expectedly less when exposed to UV light (6.8%T per °C). Furthermore, the UV transition functionality of both lenses was also demonstrated in outdoor conditions under sunlight exposure. The latter was done to assess whether the transition properties are only pertinent to a narrow range of UV light wavelengths and if the lenses are solely functional under high intensity of UV light. Yet, as shown in Fig. 5e, the transition and multifunctional contact lenses were activated under sunlight similar to their activation in response to the controlled indoor UV light. Visually, their changes were as pronounced as the ones shown in Fig. 4. This shows that these contact lenses can, in fact, respond and protect against harmful UV radiations from the sunlight, which highlights their practical application.

**Fig. 6:**
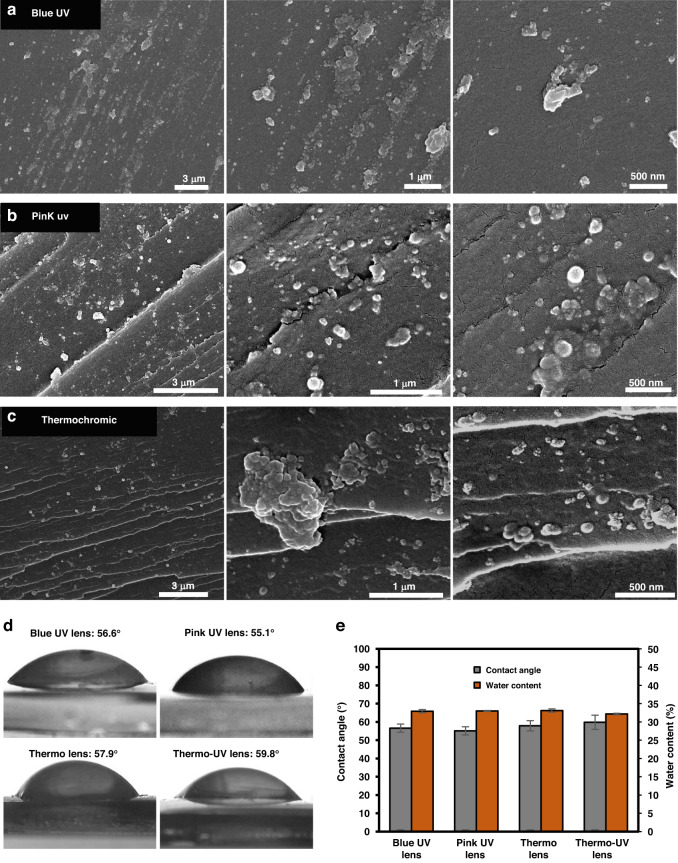
Morphological and physiochemical properties of the fabricated lenses. Cross-sectional SEM images of the fabricated lenses using (**a**) blue photochromic, (**b**) pink photochromic, and (**c**) thermochromic powders. **d** Contact angle images of the fabricated lenses, utilizing the sessile drop method with 5 µl droplet. Wettability and water retention assessment. **e** Contact angle and water content measurements (after 72 h) of the fabricated lenses, showing the average and standard error of three measurements

The morphology and distribution of the micro-powders within the lenses were evaluated through SEM imaging of the cross-section, and the resulting images at different scales are shown in Fig. 6a–c. All micro-powders seem to have retained their spherical morphology, yet their sizes (~500 nm) were much smaller compared to the solely imaged powders (Fig. 2), which indicates the powders most probably disintegrated when mixed with hydrogel solution. Moreover, the blue and pink UV powders were well distributed within the contact lenses while the thermochromic micro-powders showed more signs of cluster formation, possibly due to higher surface energy.

The physiochemical properties of the contact lenses, in particular wettability and water retention, were assessed by measuring the static contact angle using the sessile drop method and the water content over 72 h (Fig. 6d, e). Contact angle measurements were done in triplicates, and the average value for each lens was reported. The fabricated lenses were generally hydrophilic, and the contact angle values for the four lenses were in the range of 55–60°. These minimal changes indicate that both integrated powders had insignificant effects on the wettability of the lenses. The aforementioned wettability range is similar to the wettability values reported previously for HEMA hydrogels^44^. Also, the reported values are within the acceptable wettability range of commercial contact lenses (45–80°)^5^^,45^. Likewise, water content assessment is vital to ensure adequate hydration of the lenses and to get an indication on the lenses’ oxygen permeability^46^. The water content properties of the contact lenses were assessed by completely drying the lenses and measuring their water absorption capacity, 72 h after placing them in DI water. The average water retention of the four lenses were around 32–33%, with no apparent effect observed due to micro-powders’ incorporation. The measured water content capacities were also similar to the values for HEMA hydrogels reported previously and within the range of commerical products (25–50%)^44^. Accordingly, physiochemical properties among all fabricated contact lenses had minor differences. Similarly, powder concentration had no effect on the aforementioned properties, which can be noted by examining the measurement differences between the thermochromic and multifunctional contact lenses. As such, no particular trends can be established. The measured properties were all within the range of commercial contact lenses, indicating the adequacy of the contact lenses’ materials and fabrication method.

## Discussion

Multifunctional transition and temperature-responsive contact lenses were fabricated via molding technique, and their optical properties at activated/inactivated states were analyzed. Transition contact lenses showed adequate UV (~45%) and blue light (~20%) blocking capabilities under normal lightning, whereas under UV irradiation the contact lenses were responsive as they darkened and absorbed portions of the visible light spectra. The discrepancies between the blue and pink lenses allow for customization as per the user’s need. Likewise, the temperature-sensitive and multifunctional (including both transition and thermochomism functionalities) showed colorimetric and optical variations across physiological conditions (33–38 °C), and their sensitivity ranges were around 7–8% transmitted light per Celsius degree shift. Practicality of the multifunctional contact lenses was apparent as they responded (darkened) in outdoor conditions due to the UV radiation from the sunlight incidence. As such, their transition properties were analogous to the ones obtained indoor under UV illumination. Furthermore, wettability and water content of the contact lenses were not altered as a result of the incorporated functional materials. These measured physiochemical properties were within the range of commercial products, yet properties like oxygen permeability, biocompatibility, and protein deposition still need to be assessed. Finally, since these multifunctional lenses showed good UV and blue light filtering capabilities along with physiological colorimetric temperature-responsiveness, they can potentially be utilized as therapeutic and diagnostic wearable aids to monitor and prevent various ocular diseases.

## Materials and methods

### Materials

2-Hydroxyethyl methacrylate (HEMA, 97%) containing ≤250 ppm of monomethyl ether hydroquinone as inhibitor, Ethylene glycol dimethacrylate (EGDMA, 98%) containing 90–110 ppm of monomethyl ether hydroquinone as inhibitor, 2-hydroxy-2-methylpropiophenone (97%), and dimethyl sulfoxide (DMSO) were purchased and used from Sigma Aldrich as is without further purification. Photochromic and thermochromic powders were purchased from Hangzhou Tiankai, Zhejiang, China.

### Fabrication

Contact lenses were fabricated by mixing monomer (HEMA), crosslinker (EGDMA), and photoinitiator (2-hydroxy-2-methylpropiophenone) with a ratio of 95:3.5:1.5 vol%, respectively. Then, the concentration of the added powders was optimized to ensure adequate transparency and color intensity. Transition contact lenses were fabricated by using two powders, pink and blue, and adding them in three different concentrations: 0.15, 0.3, and 0.45 wt% to the polymer solution, thus yielding a total of six contact lenses. As for temperature sensitive lenses, they were synthesized using 0.3 wt% thermochromic powder that was mixed with the polymer solution. Multifunctional transition temperature sensitive contact lenses were fabricated by adding 0.3 wt% of both thermochromic and pink photochromic powders. Moreover, the concentration selection was optimized to ensure contact lenses are transparent (>80%) and have adequate coloration properties. In fact, we noticed that increasing the powder concentration beyond 0.6 wt% reduces the overall transmission significantly, which diminishes its optical transparency. A photochromic powder concentration of 0.3 wt% demonstrated viable coloration and optical transparency properties; hence, we could have only added 0.3 wt% of the thermochromic powder, so as not to go beyond the 0.6 wt% limiting concentration. The resulting solutions were mixed for 30 min until powders completely dissolved. Subsequently, they were placed in contact lens mold and UV polymerized for 10 min at 365 nm using UVP Crosslinker CL-1000L, Analytik Jena (Fig. 1b). The lenses were then washed using isopropyl alcohol and distilled water 50:50 vol%.

### Characterization

The multifunctional contact lenses were characterized for their optical, physiochemical, and morphological properties. Prior to that, thermochromic and photochromic powders were characterized using fourier-transform infrared spectroscopy (FTIR), scanning electron microscopy (SEM), and UV–Visible spectroscopy. Chemical composition of the powders was analyzed through their FTIR spectra. Attenuated total reflection (ATR) mode in the Spotlight 200 FTIR microscopy system was utilized to obtain the FTIR spectra of the powders. Scanning electron microscopy (SEM) (FEI Nova NanoSEM 650, beam resolution 0.8 nm) was utilized to image the morphology of the microcapsule powders. Powders were dissolved in DMSO 10 wt%, and then few droplets were deposited on a carbon tape, which were left to dry in an oven at 80 °C. The absorption spectra of the dissolved powders in DMSO was measured using a UV–Vis spectrometer (USB 2000+, Ocean Optics). Activated and inactivated states of the powders were measured. For the photochromic powders, active states were recorded by exposing the powdered solution to UV using a handheld torch light. For the thermochromic powder, the solution was heated till 38 °C, the saturation temperature for the powder, after which it exhibits no colorimetric variation.

Furthermore, inactive optical transmission spectra of the contact lenses were recorded using Lambda 1050 UV/Vis/NIR spectrometer. Since contact lenses cannot be exposed to UV light inside the Lambda 1050 spectrometer, the transmission of the UV active states was obtained using a customized setup. A spectrophotometer, USB 2000+, was attached to an optical microscope, and a torch light (375 nm, 1600 lumens) was turned on to expose the multifunctional transition lenses to UV light and record the corresponding response. To measure the temperature response of the thermochromic lenses, they were placed at two different temperatures (33 and 38 °C), and their corresponding spectra was measured using Lambda 1050 spectrometer. The aforementioned tests were also conducted on multifunctional transition and temperature-sensitive lenses to assess their optical responsivity at different states for each powder (active and inactive). Moreover, hydration and wettability of the synthesized contact lenses were obtained by measuring their water content and static contact angle. To measure the water content of the lenses, samples were placed in a vacuum oven at 60 °C for three hours, and their dry weight was recorded. Upon immersing them in deionized (DI) water for 72 h, their total weight was obtained, and corresponding water content was deduced. The static contact angle was measured using the sessile drop method, in which a 5 µl water droplet is deposited on the sample, and the resolved angle image is captured. The water content and contact angle measurements were done in triplicates, for which the averages and standard deviations were reported. Finally, morphologies of the lenses with thermochromic and photochromic powders were also imaged using SEM. Cross-sectional images were obtained by shearing the contact lenses, placing them on a carbon tape, and coating them with a 10 nm gold layer to avoid the charging effect.

### Supplementary information

Supplementary File
