# Supplementary material for: Multifunctional transition and temperature-responsive contact lenses
Source: Light Sci Appl. 2023 Nov 13;12:271. doi: 10.1038/s41377-023-01304-1 (PMC10643561; doi:10.1038/s41377-023-01304-1)
Supplement: Supplementary file 1 — Supplementary File [file 41377_2023_1304_MOESM1_ESM.docx]

**Supplementary Information**

Multifunctional Transition & Temperature-Responsive Contact Lenses

*Ahmed E. Salih,^a,*^ Haider Butt ^a,*^*

^a^ Department of Mechanical Engineering, Khalifa University, Abu Dhabi, UAE

*Email: [aesalih95@gmail.com](mailto:aesalih95@gmail.com), [haider.butt@ku.ac.ae](mailto:haider.butt@ku.ac.ae)


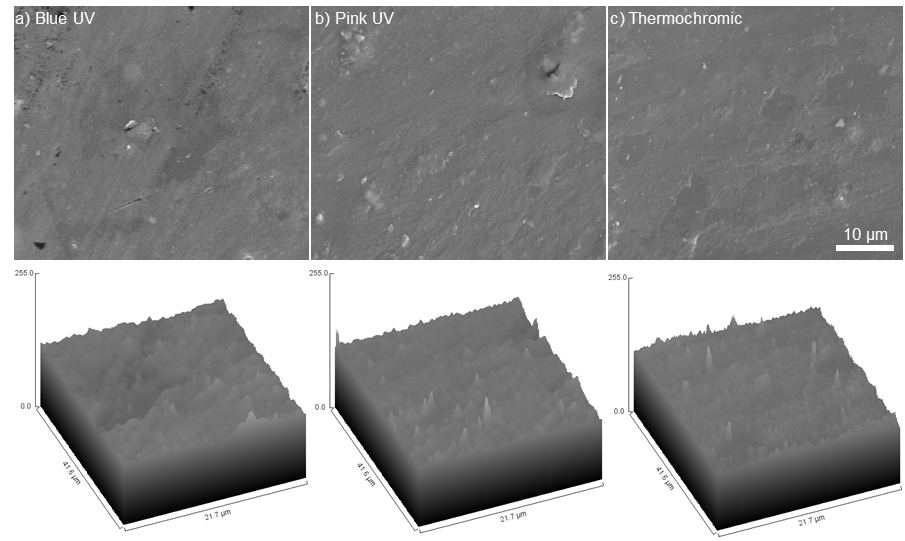


Figure S1: *SEM surface images (up) and extracted surface profile (down) using imageJ of the a) blue and b) pink photochromic and c) thermochromic contact lenses.*


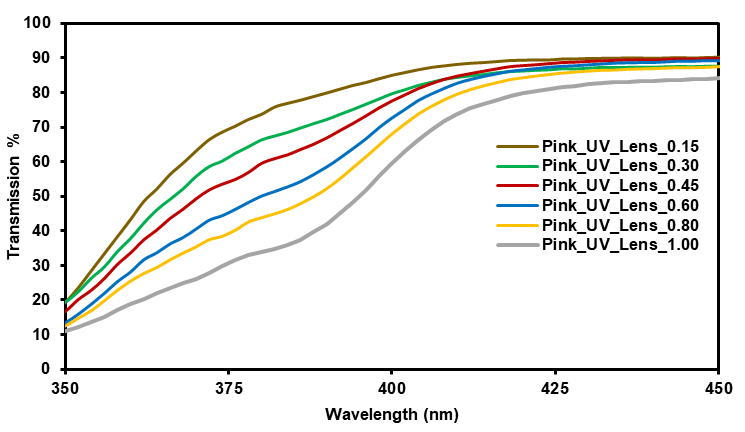


*Figure S2: Effect of pink photochromic powder concentration on the transmission spectra of the lenses and their light filtering capabilities.*
